# Nanosilica-Toughened Epoxy Resins

**DOI:** 10.3390/polym12081777

**Published:** 2020-08-08

**Authors:** Stephan Sprenger

**Affiliations:** Evonik Operations GmbH, Charlottenburger Strasse 9, 21502 Geesthacht, Germany; stephan.sprenger@evonik.com

**Keywords:** epoxy resin, silica nanoparticles, toughening, fatigue, nanotoxicity

## Abstract

Surface-modified silica nanoparticles are available as concentrates in epoxy resins in industrial quantities for nearly 20 years. Meanwhile, they are used in many epoxy resin formulations for various applications like fiber-reinforced composites, adhesives or electronic components; even in space vehicles like satellites. Some of the drawbacks of “classic” epoxy toughening using elastomers as a second phase, like lower modulus or a loss in strength can be compensated by using nanosilica together with such tougheners. Apparently, there exists a synergy as toughness and fatigue performance are increased significantly. This work intends to provide an overview regarding the possibilities of nanotoughening with silica, the industrial applications of such epoxy resin formulations and the most recent research results.

## 1. Introduction

For many years epoxy resins are used in a continuously growing variety of industrial applications. From aircraft components to high-performance automotive adhesives, from rotor blades for wind energy installations to heavy-duty coatings for the marine industry. They are used in electronic components like automotive sensors or mobile phone cameras as well as in pipes for offshore oil platforms.

However, the number of epoxy resins manufactured and used in industrial quantities remained relatively constant over the last 30 or more years. The diglycidyl ether of bisphenol A (DGEBA) is used by far in the largest volumes and represents the working horse of the industry. Second in volume is the diglycidyl ether of bisphenol F (DGEBF). Short-chain aliphatic epoxy resins, i.e., hexanediol diglycidyl ether (HDDGE), are sometimes added to reduce viscosities and increase flexibility. Multifunctional epoxy resins like the triglycidyl ether of aminophenole (TGEAP) or tetraglycidyl ether of methyl dianiline (TGMDA) are designed for high-performance aerospace applications. Epoxidized soya bean oil or cashew nut shell oil are cheap flexibilizers for coating applications. Cycloaliphatic epoxy resins, i.e., epoxy cyclohexylmethyl epoxy cyclohexane (EEC), are the main ingredient in UV-curable formulations like stereolithographic resins (SLA).

The number of hardeners is constantly increasing. They are defining not only the mechanical, chemical and thermal properties of the cured epoxy resin by forming the three-dimensional molecular network upon cure, they also define the cure schedule by cure temperature and cure speed. This is a very important criterion for the processing conditions of the epoxy resin: one-component systems, two-component systems, room temperature cure, elevated temperature cure, radiation cure, fast cure, slow cure, postcure or not.

Furthermore, they can also have a strong influence on the viscosity: in paste adhesives solid, curing agents are preferred, whereas in coatings, low viscosity liquids are more suitable. Most curing agents are amine-functional, from simple low molecular primary amines over long, flexible polyamines to aromatic amines or sterically hindered aromatic amines.

Amides, acid anhydrides, cationic curing agents and catalytic curing agents are used as well, sometimes together with UV initiators. Formulating epoxy curing agents is an art and requires considerable knowledge, hence many companies keep this knowledge confidential.

Besides the hardener, other formulation ingredients have a tremendous influence on the property profile of the cured epoxy resins, i.e., fillers to improve mechanical properties, additives to reduce flammability, fillers to increase heat transfer, additives to improve adhesion to a variety of different substrates and additives to change electrical properties.

The real Achilles heel of epoxy resins is their inherent brittleness. Consequently, over the years a variety of tougheners, reactive as well as nonreactive, have been developed. Some technologies are established in many industrial applications, others have been designed for specialty applications only. It is well known that fillers like clay, fumed silica or short-cut glass fibers can increase toughness as well and often perform synergistically with elastomeric tougheners. 

With nanoscaled additives becoming more and more available and affordable for industrial applications, they also became the subject of intensive research. Fu and Li published recently an overview regarding the nanomodification of polymers [1]. A much more detailed, very extensive review was elaborated by Hadavinia et al. [2]. They focused on the available insights of the property improvements of epoxy resins achieved by the modification with various nanomaterials.

**Figure 1:**
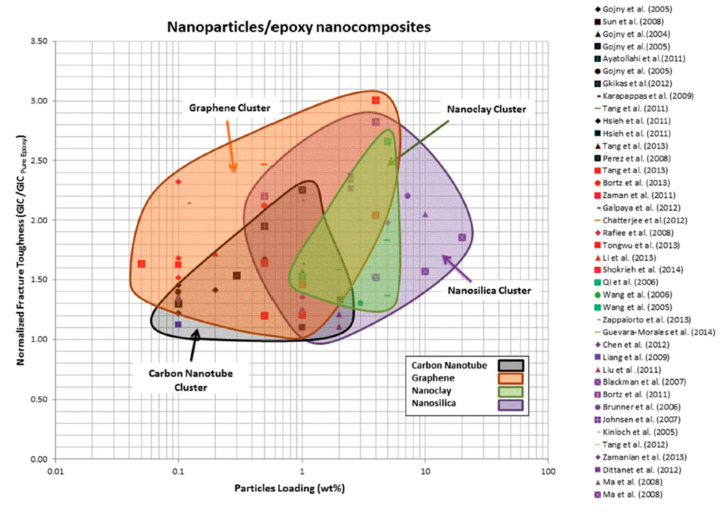
Map of fracture toughness of nanoparticles/epoxy nanocomposites with respect to particle loading [2].

It is interesting to see in Figure 1 that the biggest increase in toughness can be achieved with the addition of graphene as well as with the addition of nanosilica, both at addition levels around 4 wt %. However, of course, graphene is outperformed by nanosilica when it comes to strength and modulus. Other nanomaterials like nanoclays or carbon nanotubes seem to be less efficient as tougheners for epoxy resins at similar addition levels. As for any industrial material, the price increase of the final product by the modification needs to be justified by the improvements achievable. Furthermore, the ease of handling in industrial applications has to be taken into account, the viscosity of the modified resin becomes quite important (compare Figure 4 and especially 3.4 and 3.5), which is i.e., a big issue for graphene-modified epoxy resins. Considering all this, it is not very astonishing that the most widespread nanomodification of epoxy resins used in large volumes in many different applications is the addition of nanosilica.

**Figure 2:**
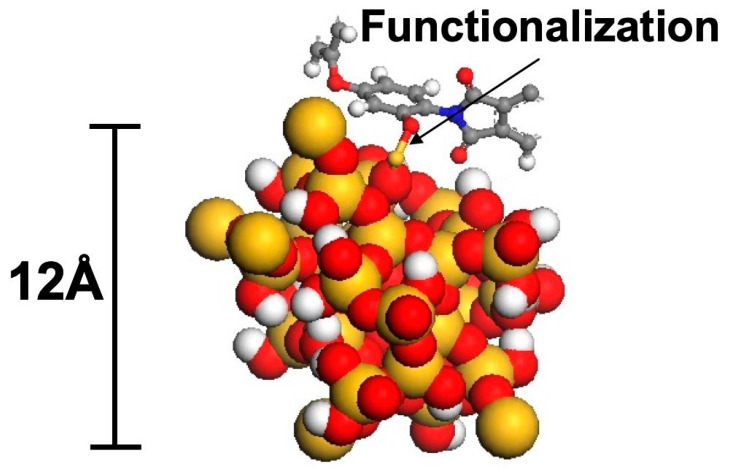
Molecular model of a functionalized silica nanoparticle [3].

Silica nanoparticles are commercially available for 20 years. They are surface-modified in order to suppress agglomeration and to increase the compatibility to the matrix as well as to enable bonding to the matrix upon cure. In Figure 2, the model of such a particle with a size of approx. 1.2 nm, calculated by Odegard et al. [3], is shown. The huge number of hydroxy groups on the surface of the particle, which are subject to chemical surface modification, is notable. They are extremely important for the intensive interaction with the epoxy resin matrix and the mechanisms of nanotoughening (compare Section 2.3).

**Figure 3:**
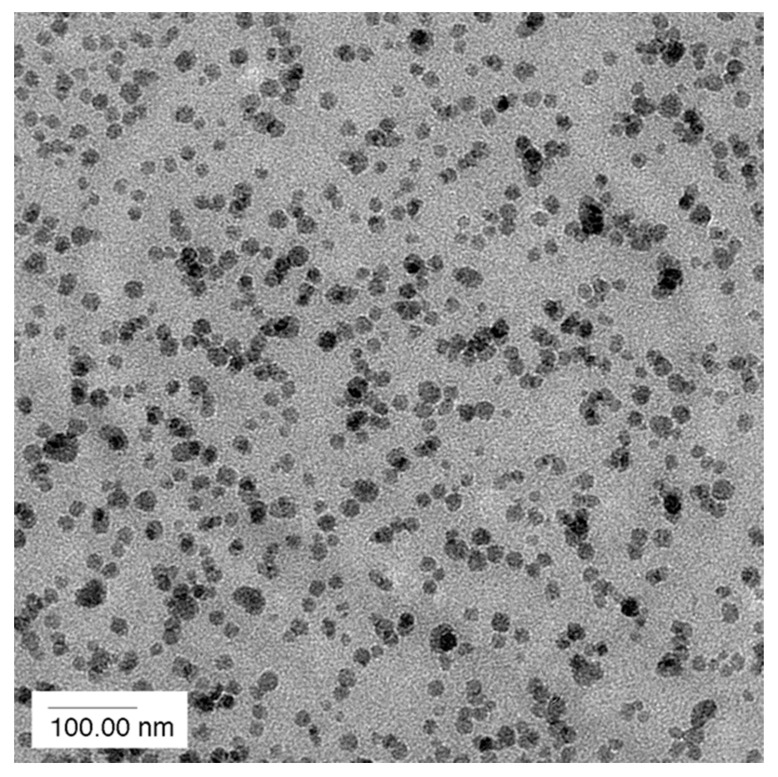
Transmission electron microscopy picture of an epoxy resin with 5 wt % nanosilica [5].

Commercially available silica nanoparticles are generally manufactured by a sol–gel process and have a particle size of approx. 20 nm. They have a spherical shape and a very narrow particle size distribution, as can be seen in Figure 3 (for further details see [4]).

The researchers cited in this work have been using commercially available 40 wt % concentrated masterbatches of surface-modified silica nanoparticles in various epoxy resins. Within these concentrates, the nanoparticles are monodispersed. To vary the nanosilica concentration, they are diluted with standard epoxy resins. Using the commercially available masterbatches has tremendous advantages because the silica nanoparticles do not agglomerate upon further dilution of these concentrates, as can be seen in Figure 3. Even blending them with epoxy resins of different chemistry, i.e., mixing a DGEBA-based nanosilica concentrate with TGMDA, does not create any agglomerates. Furthermore, simple stirring is sufficient for blending in order to obtain well-dispersed nanoparticles. No complex mixing procedures or high sophisticated equipment are necessary. This might explain why nanosilica in a pure form, as powder, became no commercial success for epoxy resins.

Being spherical, they do not exhibit any thixotropic properties, as larger, branched structures of silica typically do. The resins containing such silica nanoparticles behave like Newtonian liquids. In most cases, uniform distributions of the nanoparticles were found in microscopical investigations, regardless of the type of epoxy resin and hardener used. In a few cases the formation of agglomerates was reported, always caused by incompatible additives used in the epoxy resin formulation or by large amounts of only partially compatible tougheners. Agglomeration does negatively affect the property improvements obtained by the nanosilica addition and should be avoided by designing the epoxy resin formulation accordingly.

**Figure 4:**
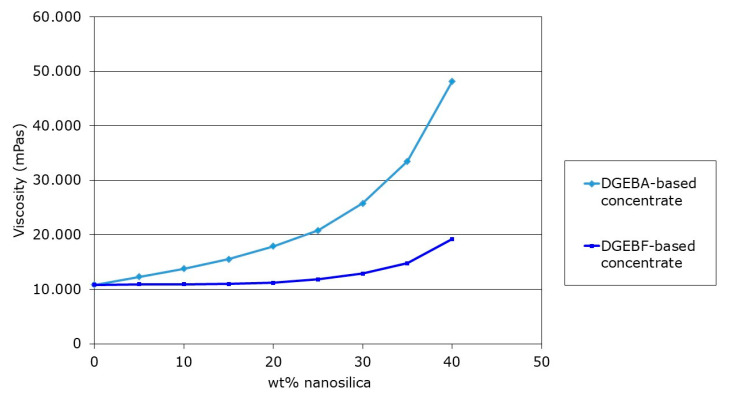
Viscosity increase of a diglycidyl ether of bisphenol A (DGEBA) epoxy resin with increasing nanosilica content using different nanosilica concentrates [4].

At typical addition levels of 8–10 wt %, they do increase the resin viscosity only insignificantly and can be easily handled in production equipment, as can be seen in Figure 4.

## 2. Epoxy Resins Modified with Silica Nanoparticles

### 2.1. Toxicological Aspects of Silica Nanoparticles in Epoxy Resins 

When talking about the use of nanoscaled structures, it has to be accepted that the term “nano” has often a negative connotation for the general public. Such discussions are usually conducted with a lot of emotions but no or little expert knowledge and are far from a scholarly debate. Hence, it is important to have a closer look at the toxicological aspects of silica nanoparticles. Although an overarching elaboration would go beyond the scope of this work, some fundamental aspects need to be covered.

First, it has to be mentioned that the silica nanoparticles obtained by sol–gel processes are amorphous, with no crystalline fractions, which could be shown by small-angle neutron scattering investigations. After decades of experience with the industrial production and application of amorphous silica, no negative effects on humans have yet been observed. By oral uptake, amorphous silica is not toxic; colloidal silica sols are used in many applications in the food industry, i.e., to clarify fruit juices. 

Very important as well is the aspect ratio. The commercially available silica nanoparticles are spherical (compare Figure 2 and Figure 3). This makes them no threat to macrophages, as rod-like structures with a high aspect ratio (i.e., asbestos or, much smaller, of course, carbon nanotubes) are.

An investigation published by the German Federal Institute for Occupational Safety and Health (BAUA) regarding the genotoxicity of ultrafine dust in lungs probed nanoparticles of different varieties: crystalline silica, amorphous silica, titanium dioxide, carbon black and fullerenes [6]. For amorphous silica nanoparticles no genotoxicity was reported. Dawson and Howard et al. confirmed that no genotoxicity of amorphous nanoparticles of various sizes (between 21 and 240 nm) was found regarding T3T cell systems [7].

A study dedicated to amorphous silica nanoparticles, based on in vitro and in vivo results, published by Fruijtier-Poelloth, came to the conclusion that silica nanoparticles have no different toxicity than larger particles [8]. No bioaccumulation or biopersistence was reported, the silica nanoparticles disappeared from living organisms rather quickly by physiological excretion mechanisms. 

A very extensive review covering both crystalline and amorphous silica nanoparticles was done by Hoet et al. [9]. They investigated the mechanisms of potential toxicological action and collected a huge number of test results from both in vitro and in vivo tests. They stated that synthetic amorphous nanosilica is not contaminated with crystalline silica and hence less “dangerous” than naturally occurring amorphous silica. However, they concluded that still not enough data is available to grant a waiver for all silica nanomaterials. A recent survey on the state of nanosafety research in Europe and the US confirmed the need for more investigations [10].

Therefore it is extremely important to avoid consequently any contact or exposure to isolated silica nanoparticles. The industrial particles available have been designed in such a way: during the entire manufacturing process as well as in the use of the product and its entire life cycle no isolated particles occur. In sol–gel manufacturing methods, as currently used, the nanoparticles are always trapped in a matrix, first in water, then in solvents and finally in an epoxy resin. The commercial products, concentrates of silica nanoparticles in epoxy resins, are easy to handle liquids and easy to blend with other epoxy resins. The safety measurements necessary to handle epoxy resins are sufficient. Testing of the air in the workplace using special nanoparticle detectors did not reveal the presence of any isolated particles. 

After curing the epoxy resin formulation, the particles are immobilized within the resin matrix and covalently bond to the epoxy resin. Investigations of the dust formed by mechanical treatment of nanosilica containing cured epoxy resins with various abrasives did not show the presence of any isolated nanomaterial. Instead, larger polymer fragments with the nanosilica particles still embedded were found. 

The next question to be answered is what will happen in case of a fire or a thermal decomposition of the cured epoxy resin, when the polymer matrix around the nanoparticles disappears. Will there be isolated silica nanoparticles in the smoke, the fire effluents or in the ash? No isolated nanoparticles were found, but larger structures or agglomerates similar to fumed silica [11,12]. Such micron-sized agglomerates will be easily absorbed by conventional air filter systems. Apparently, in parallel to the thermal decomposition of the cured epoxy resin matrix the organic coating of the particles is affected as well, which leads to the immediate formation of the larger agglomerates similar to fumed silica. This is extremely important not only in case of an accidental fire, but regarding the fact that most epoxy resin-based materials are recycled currently thermally in steel or glass manufacturing.

### 2.2. Property Improvements of Epoxy Resins by Silica Nanoparticle Modification

When silica nanoparticles have been introduced into epoxy resin applications more than two decades ago, the first intention was to have a functional filler that does not affect the viscosity of the epoxy resin. Conventional, macro scaled fillers increase viscosity significantly, which prevents their use in many formulations.

Being that small, the SiO_2_ nanoparticles do not interfere with the visible light and appear transparent. This is very important for all radiation cured systems, like cycloaliphatic epoxy resins used in stereolithography. It is important as well in fiber-reinforced composites applications, when the parts are not painted, but supposed to show the structure of the fabric used, as it is often the case for automotive parts made from carbon fibers. Of course, silica nanoparticles can be used in infusion or injection processes, as they are capable of penetrating even close-meshed fabrics without being filtered out, as would be the case with conventional, micron-sized filler particles.

The size is very important as well when it comes to many electrical and electronic applications, like flip-chip, chip underfill or encapsulation. It was found that electro corrosion of encapsulated generators can be reduced, as the nanoparticles are capable of penetrating between the copper wires, even when the generators are extremely large. This translates into a much longer service life of the generator.

The silica nanoparticles do not affect the glass transition temperature (T_g_) at low addition levels below 10 wt %; at higher addition levels, a slight reduction by 2–5 °C can be observed.

**Figure 5:**
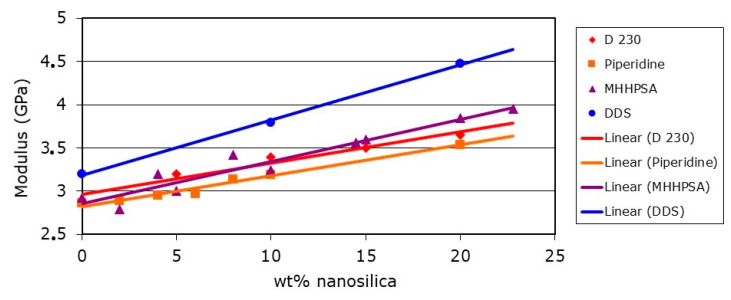
DGEBA epoxy resin modulus as a function of silica nanoparticle content for different curing agents [13].

Of course the use of a filler, no matter what size, always increases the modulus of a cured epoxy resin. Figure 5 shows the modulus increase as a function of the silica nanoparticle content for a DGEBA standard epoxy resin.

Obviously, depending on the chemical nature of the curing agent and its influence on the network formation, the modulus of the cured unmodified DGEBA is different. Diaminodiphenyl sulfone (DDS) provides the highest values, followed by methylhexahydrophthalic acid anhydride (MHHPSA), polyether amine (D 230) and piperidine. The further increase in modulus of the cured resin with increasing addition levels of silica nanoparticles is linear. 

The tensile strength seems not to be increased by the addition of nanosilica, although some researchers report slight improvements [13]. Probably the chemical nature of the curing agent has a larger influence on tensile strength than on the modulus. 

Compressive strength and compressive modulus are increased by up to 30% at a 10 wt % addition level of nanoparticles. Jumahat et al. [14] reported larger increases for tensile strength, tensile modulus, compressive strength and compressive modulus as well as a linear function between property improvement and nanosilica addition level. 

Zhang et al. investigated the strain-rate effect on compressive properties of a highly crosslinked tetrafunctional epoxy resin, the tetraglycidyl ether of methyl dianiline (TGMDA) cured with diamino- diphenyl sulfone (DDS) [15]. Then, 10 wt % nanosilica were added by using a commercial 40 wt % concentrate in DGEBA. They reported an increase in compressive modulus which they ascribed to the much higher modulus of silica compared to an epoxy resin. The strength improvements found were ascribed to the uniform distribution of the nanoparticles in the resin which may reduce local stress concentrations causing premature failure. The improvements were larger at lower strain rates and became smaller at higher strain rates, probably due to the reduced interactions between nanosilica and epoxy resin matrix. Dmitriev et al. used an amine-cured DGEBA with a much lower crosslink density to obtain the experimental data when modeling the stress-strain behavior [16]. When assuming an increase in strength by the nanosilica addition they obtained a good correlation between experimental and modeling results. They attributed the increase in strength to an increased crosslinking but provided no supportive data, i.e., T_g_ values.

A very extensive modeling study regarding the stiffness improvements from nanosilica addition was provided by Thorvaldsen et al. [17]. They found good agreement between their theoretical models and the experimental data obtained from DGEBA cure with an anhydride hardener as well as with an amine curing agent. Stiffness predictions in the function of the nanosilica addition level became possible.

Another interesting property improvement achieved by the addition of nanosilica was reported by Zhang et al. [18]. They investigated the wear and friction properties of an anhydride-cured DGEBA containing wax microcapsules and nanosilica. Optimal tribological properties with a reduction in wear rate of three orders of magnitude and a tenfold reduction in the coefficient of friction were found for 10 wt % wax microcapsules in combination with 23 wt % of silica nanoparticles.

Zhang et al. investigated the wear properties of amine-cured DGEBA with 2 wt % SiO_2_ nanoparticles in a marine environment [19]. The immersion in saltwater decreased toughness for both the unmodified and the modified epoxy, but after 30 days, the nanosilica containing epoxy still had the fracture toughness of the unmodified system before immersion. The coefficient of friction was superior as well.

When cured bulk epoxy resin systems are subjected to cyclic loading, the so-called fatigue performance is improved by the addition of silica nanoparticles as well. Mai et al. tested an amine-cured DGEBA and found an improvement of 1.5 times at an addition level of 2 wt %, but less improvement at higher addition levels [20]. In contrast Kinloch et al., investigating anhydride-cured DGEBA, could show that the improvement in fatigue performance did increase with increasing the addition level of nanosilica [21]. Further fatigue testing was done in epoxy resin applications like fiber-reinforced composites and adhesives and will be discussed together with the underlying mechanisms in Section 2.3.

Another aspect of using inorganic fillers and, of course, nanosilica in epoxy resins is the reduction of the coefficient of thermal expansion by replacing organic material with inorganic material. Investigations with various epoxy resins and different hardeners showed a decrease in the coefficient of thermal expansion (CTE) with increased SiO_2_ content [13].

With the mass production of fiber-reinforced composites parts, i.e., for automotive applications, the speed of cure becomes an important factor for the economic efficiency of manufacturing processes. The modification of the epoxy resins used with silica nanoparticles does not only improve the mechanical performance, but can reduce the exotherm. Taylor et al. were able to show that the curing reaction itself was not influenced but the total heat of reaction was reduced [22,23]. Furthermore, they were able to develop a kinetic and rheological model that enables them to predict and control fast cure cycles and/or manufacture thick parts.

### 2.3. Toughness Increase of Epoxy Resins by Nanosilica Modification and Mechanisms

As already mentioned several times, epoxy resins are inherently brittle. Hence, the interest in tougheners is as old as epoxy resin chemistry itself. When nanoparticles as modifiers came up, much of the early research work was dedicated to the toughness increase of epoxy resins [13]. 

**Figure 6:**
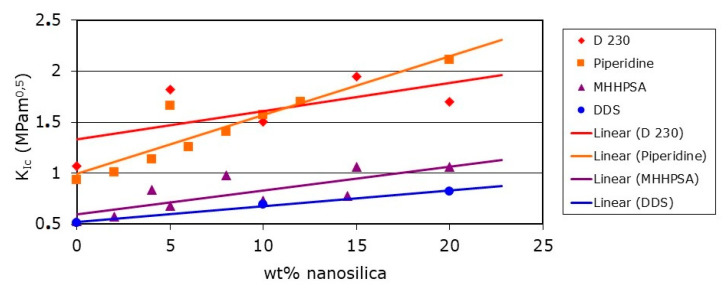
DGEBA epoxy resin fracture toughness using K_Ic_ as a function of silica nanoparticle content for different curing agents [13].

In Figure 6, an overview of the increase of the critical stress intensity factor (K_Ic_) as an indicator for the fracture toughness of modified epoxy resins is given. The interested reader can find detailed tables with values obtained from various sources including error bars and further detailed information in [13]. 

Hardeners providing a high crosslink density like DDS or MHHPSA yield of course a cured epoxy resin with a lower toughness, as brittleness increases with increasing crosslink density. Moreover, it becomes evident that for flexible hardeners like D 230 there is no linear correlation between toughness increase and increased addition level of silica nanoparticles, but an optimal addition level does exist.

In many industrial formulations for various applications the optimal addition level is not chosen by the maximal toughness achievable, but for the maximal improvements of various properties important for the respective application, including price.

Compared to classical rubber toughening of epoxies, which can increase toughness by factor two to four, the toughness increase obtained with silica nanoparticles at typical addition levels of 10 wt % is rather a 50% increase. Rein et al. reported the G_Ic_ of an anhydride-cured blend of DGEBA and hexanediol diglycidyl ether (HDDGE) to be doubled when 24 wt % nanosilica is added [11]. Moeller et al. tested organophosphorus modified epoxy novolac resins in combination with silica nanoparticles regarding their flame retarding properties and reported an increase in G_Ic_ at 4.7 vol % (approx. 9.4 wt %) SiO_2_ [12].

Xu et al. compared the toughening performance of 6 wt % nanosilica with 6 wt % of a core shell rubber Type I (nomenclature from [24]) having an average diameter of 100 nm [25]. In an amine-cured DGEBA both provided similar fracture toughness increases at −50 °C, but no increase at 80 °C. At ambient temperatures, the core shell nano rubber showed no increase in fracture toughness, whereas the toughness of the nanosilica-modified epoxy resin was improved by 25%.

Tsang compared silica nanoparticles and very large elastomeric core shell particles Type II (38–106 µm) in an anhydride-cured DGEBA [26]. At a 10 wt % addition level, the nanosilica doubled the fracture energy, whereas the core shell modification nearly quadrupled the fracture toughness.

The mechanisms of toughening with classical, micron-sized fillers are well understood for many years and many different polymer systems, both thermoplastic and thermosetting. A very high-stress concentration, caused by mechanical impact, external tensions or inner tensions upon cure, normally leads to a crack formation and consequently to a failure of the thermosetting polymer, as the molecular network is destroyed. Insights in the property-morphology relationship of thermoplastic materials modified with nanoparticles and especially nanosilica were not helpful when investigating epoxy resins: the three-dimensional molecular network is formed irreversibly upon cure when the epoxy resin reacts with the hardener. DGEBA monomers have a size of approx. 2 nm, an amine hardener (i.e., isophorone diamine) is about half the size. The silica nanoparticles discussed have a size of approx. 20 nm, thus an interaction on a molecular scale between filler and thermosetting polymer needs to be understood. 

**Figure 7:**
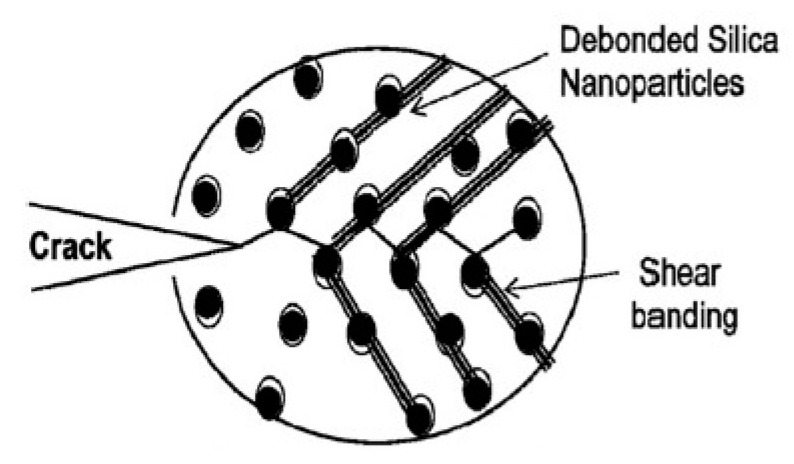
Schematic representation of plastic void growth and matrix shear banding mechanisms observed involved in the fracture of nanosilica-modified epoxy resins [29].

It is known for many years that hard glass microspheres can toughen thermoset resins [27]. Kinloch et al. proposed a similar toughening mechanism for spherical nanoparticles in cured epoxy resins as matrix [28]: upon high stress on molecular-scale partial debonding of the polymer from the particle occurs and the debonding is followed by a subsequent plastic void growth of the epoxy matrix. Localized plastic shear bands in the polymer initiated by the stress concentrations around the interphase of the silica nanoparticles contribute as well. These two major toughening mechanisms are illustrated in Figure 7. Dittanet and Pearson confirmed the proposed toughening mechanisms in their very extensive study [29]. They investigated the influence of the nanoparticle size on toughening as well and found it negligible (comparison of particle sizes 23 nm, 74 nm and 170 nm).

In continuation of their earlier work, Kinloch et al. developed a model that showed excellent agreement between the predictions and the experimental data obtained from an anhydride-cured DGEBA with various addition levels of nanosilica [30].

**Figure 8:**
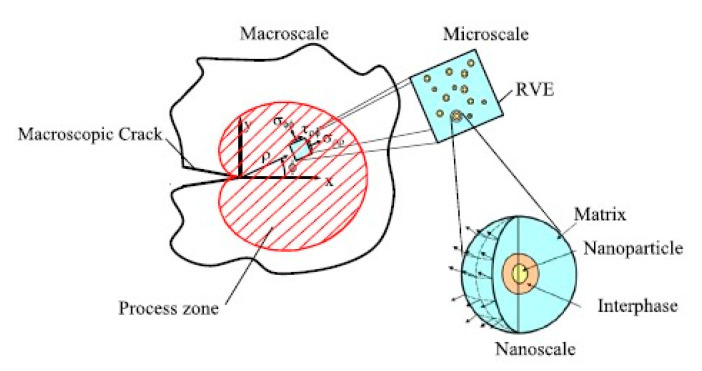
Description of the multiscale system for the assessment of the fracture toughness of nanoparticle filled polymers [32].

Zappalorto et al. used as a multiscale system (compare Figure 8) for the assessment of the fracture toughness of nanosilica in epoxy resins and found good agreement between the predictions of the model and the experimental data provided by testing an amine-cured DGEBA [31]. They compared a room temperature cure schedule with a room temperature cure followed by a 60 °C postcure and found the thermal aftertreatment to increase both strength and toughness of nanosilica-modified epoxy resins.

In extension of this work, Quaresimin et al. studied layered nanofillers like nanoclays or graphene, rod-like structures like carbon nanotubes and spherical particles using the same multiscale approach, confirming the basic toughening mechanisms again [33].

### 2.4. Synergy Between Elastomeric Tougheners and Silica Nanoparticles: Further Property Improvements

**Figure 9:**
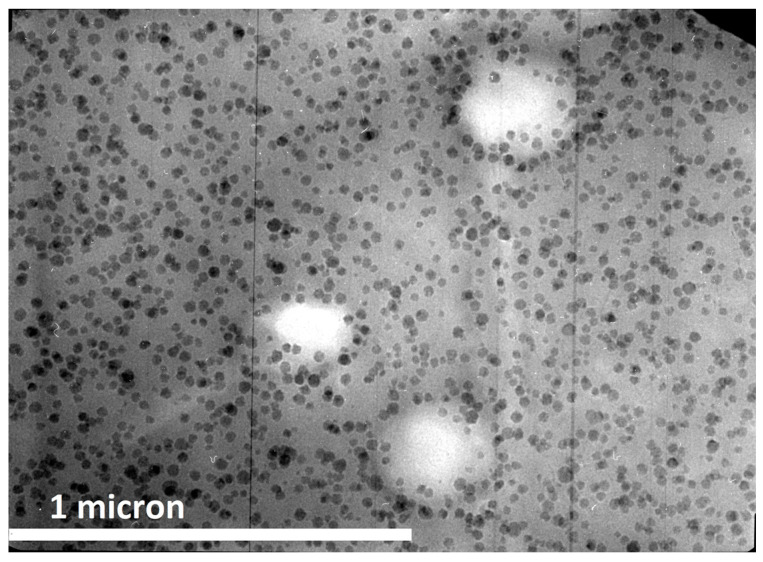
TEM picture of DGEBA modified with reactive liquid rubber (CTBN) and nanosilica [24].

**Figure 10:**
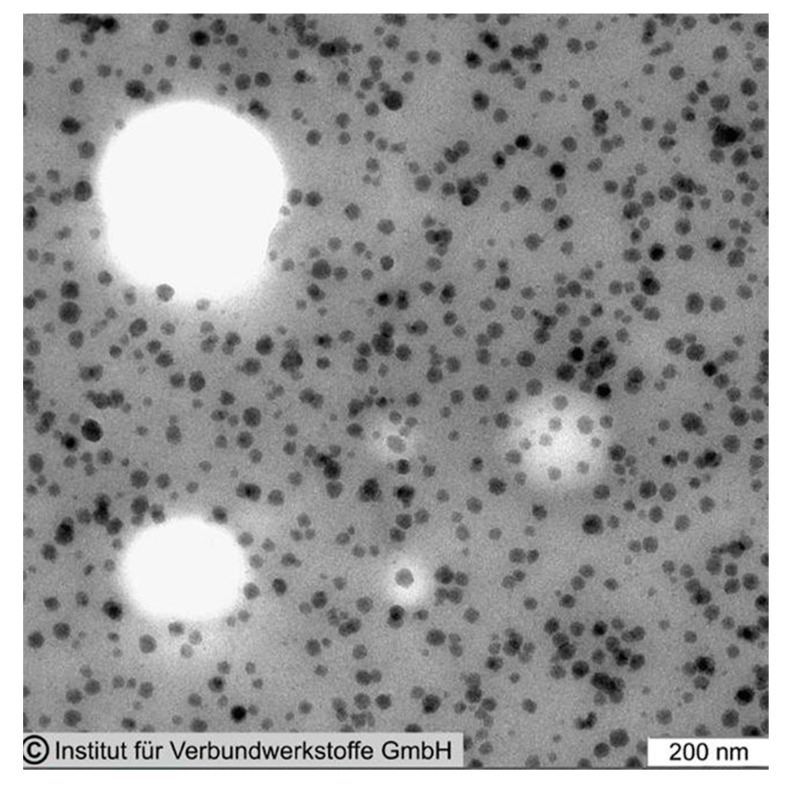
TEM picture of DGEBA modified with core shell rubber (CSR Type III) and nanosilica [24].

Very soon after the introduction of silica nanoparticles it became evident that the combination of classical elastomeric tougheners with nanosilica results in epoxy resin formulations with outstanding performance [34]. A systematic investigation revealed that the chemical nature of the elastomeric toughener is less relevant, as long as the morphology of the modified system is similar [24]. Of course, the additional levels of micron-sized elastomeric toughener and silica nanoparticles depend on the property improvements sought after and the intended use of the modified epoxy resin. Furthermore, the cure cycle has some influence as well. In Figure 9 and Figure 10, the transmission electron microscopical (TEM) pictures of two different systems are shown; although in Figure 9, an NBR rubber is used and in Figure 10 a polysiloxane rubber, the morphology looks identical. 

In an overview collecting data from both amine and anhydride-cured epoxy resin modified with combinations of either nanosilica and reactive liquid rubber (CTBN) or nanosilica and core shell elastomers (CSR Type I–III), tremendous improvements in fracture toughness were reported [24]. The G_Ic_ was increased up to 10 times, whereas the loss in modulus due to the addition of the elastomeric tougheners was very limited; the worst case reduced by 20%.

Dittanet et al. studied an amine-cured epoxy resin modified with reactive liquid rubber (CTBN), silica nanoparticles and a combination of both modifications [35]. The modulus of the cured unmodified epoxy was lowered by the addition of 15 wt % CTBN from 3.5 GPa to 2.7 GPa. Adding 5 wt % of nanosilica increased the modulus to 3.9 GPa. The hybrid system containing 15 wt % CTBN and 5 wt % SiO_2_ exhibited a modulus of 2.7 GPa. G_Ic_ was increased from 0.28 kJ/m^2^ to 2.50 kJ/m^2^ (15 wt % CTBN) or 0.66 kJ/m^2^ (5 wt % SiO_2_). The hybrid however had a fracture toughness of 3.39 kJ/m^2^, this clearly shows the synergy between micron-scaled and nanoscaled tougheners.

Ye et al. investigated amine-cured DGEBA as well, using various amine hardeners to obtain different crosslinking densities, which affects the toughening efficiency of the different modifications [36]. In their study combinations of CTBN/spherical silica nanoparticles and CTBN/halloysite nanoparticles were compared. They reported increases in G_Ic_ for epoxy resins containing 10 wt % CTBN and 10 wt % nanosilica by 138%, 298% and 450%. The hardener leading to the system with the highest initial toughness also showed the biggest improvement. This confirms that toughening of epoxy resins becomes more efficient with an increasing ductility (or toughness) of the unmodified matrix. They attributed the increases in fracture toughness mainly to voiding around the nanoparticles and shear banding as mechanisms.

Kinloch and Taylor used an anhydride-cured DGEBA pretoughened with a reactive diluent (hexanediol diglycidylether, HDDGE) to be modified with a core shell toughener Type III (average particle size 500–700 nm) and silica nanoparticles [37]. They used several models to predict elastic properties like modulus and toughness and reported very good agreement between theoretical and experimental data. The epoxy resin modified with 8 wt % nanosilica and 8 wt % core shell particles performed best: G_Ic_ was increased from 0.17 kJ/m^2^ to 1.22 kJ/m^2^. DGEBA without HDDGE exhibited a much smaller improvement in fracture toughness upon modification.

Tsang et al. based their study of an anhydride-cured DGEBA on a combination of silica nanoparticles and a core shell rubber Type II [38]. Fracture behavior at −80 °C, −40 °C and ambient temperature was tested. Again debonding followed by void growth and shear banding were confirmed as toughening mechanisms for the nanoparticles, theoretical and experimental data were matching. The core shell particles showed the classic mechanisms of rubber toughening established by Kinloch many years ago, but seemed to be not very efficient; apparently due to the agglomerated nature of the core shell particles. The fracture toughness of the hybrid system modified with 10 wt % nanosilica and 10 wt % elastomer was identical with the SiO_2_-only modified resin (G_Ic_ + 150%).

Recently a broad variety of different epoxy resin tougheners were evaluated in an amine-cured DGEBA by Doering et al. [39]. K_Ic_ of the neat resin was increased from 0.72 MPam^1/2^ to 0.96 by the addition of 5 wt % of a self-organizing block copolymer. Further addition of 5 wt % silica nanoparticles yielded the toughest hybrid system with 1.15 MPam^1/2^. An epoxy resin modified with 5 wt % core shell rubber Type III showed a similar increase to 0.93 MPam^1/2^, but was improved by 5 wt % nanosilica only to 0.99 MPam^1/2^.

In continuation of her earlier work, Dittanet et al. investigated the combination of silica nanoparticles and epoxidized natural rubber in an amine-cured DGEBA [40]. The best performance was found for the combination of 7.5 wt % epoxidized natural rubber and 5 wt % nanosilica. K_Ic_ was increased from 0.88 MPam^1/2^ to 2.21 MPam^1/2^ and G_Ic_ improved from 0.28 kJ/m^2^ to 1.80 kJ/m^2^. Modulus improved slightly from 2.25 GPa to 2.31 GPa. Although the epoxy resin modified with only 7.5 wt % epoxidized natural rubber seemed to be somewhat tougher (K_Ic_ 2.04 MPam^1/2^ and G_Ic_ 2.01 kJ/m^2^), its modulus was much lower: only 1.84 GPa. Again, the hybrid system exhibited the best balance of properties, probably the reason why such formulations became very popular in many industrial applications. 

## 3. Improving Epoxy Resin Applications with Silica Nanoparticles

### 3.1. Adhesives

**Figure 11:**
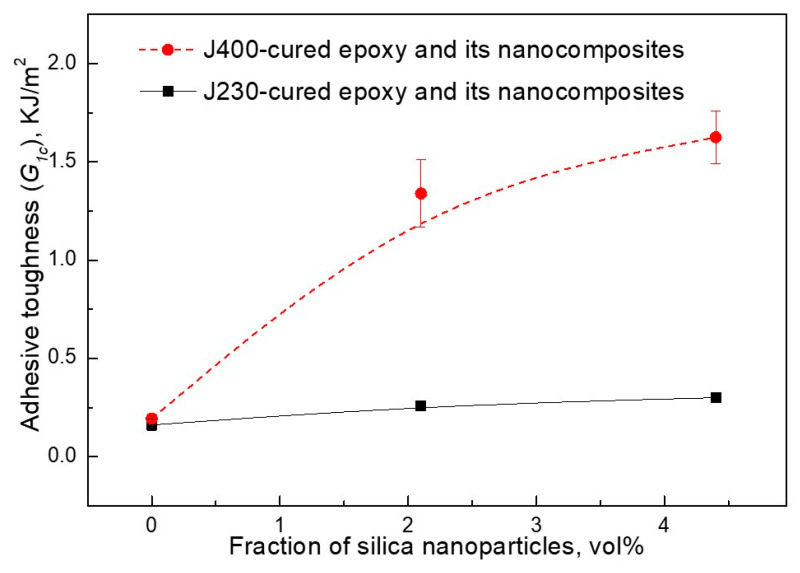
Toughness increase of epoxy adhesives by the addition of nanosilica for a brittle (J230) and a ductile (J400) adhesive [42].

The first improvements of the adhesive performance of structural epoxy adhesives were reported in 2003 by Kinloch et al. [41]. Ma et al. investigated the adhesive bonding of aluminum 6060 by double cantilever beam testing which provides toughness data [42]. DGEBA as epoxy resin was cured with two different amines (short and long-chain polyether diamines, called J230 and J400) to obtain a rather rigid and a rather ductile adhesive with a bond line thickness of 0.6 mm. In Figure 11, the toughness improvements for additional levels of 2.1 vol % (approx. 4.3 wt %) and 4.4 vol % (approx. 8.8 wt %) silica nanoparticles are shown. It is evident that the toughness improvements are significantly larger for the more ductile adhesive matrix (curing agent J 400). The brittle adhesive achieved a toughness of 0.301 kJ/m^2^, which corresponds to an improvement of approx. 60%. The more ductile adhesive however was improved by 738% from 0.194 kJ/m^2^ to 1.625 kJ/m^2^.

They used micron-sized silica particles with an average particle size of 3.2 µm for comparison and found more or less the same small improvements in toughness for both hardeners of approx. 50%. Obviously, an interaction on molecular-scale leads to a fracture surface phenomenon: TEM investigation showed the formation of a zone near the crack tip to be responsible for the impressive improvements in G_Ic_ by the nanoparticles.

Liu and Mai et al. [43] were using stainless steel as substrate for their adhesive trials, as they investigated aging under hot/wet conditions as well. They used a model adhesive consisting of DGEBA crosslinked with an amine curing agent and a bond line thickness of approx. 0.28 mm to prepare the lap shear test specimen. The lap shear strength was increased from 17.8 MPa to 21.3 MPa by the addition of 10 wt % nanosilica. A 20 wt % addition level showed no further improvement (21.5 MPa), further addition of nanosilica did reduce the strength. The adhesive modulus increased with increasing nanoparticle addition level, whereas the T_g_ was reduced minimally. Exposing the lap shear test coupons to 100% relative humidity at 60 °C reduced the strength over exposure time, with the nanosilica-modified adhesives always performing better than the control. Furthermore, they tested the cyclic loading until failure and found significant improvements for the system with 10 wt % silica nanoparticles; using 20 wt % did not result in further improvements. After the hot/wet aging of the test specimens all adhesives performed in a similar manner; the benefit of using nanoparticles disappeared.

**Figure 12:**
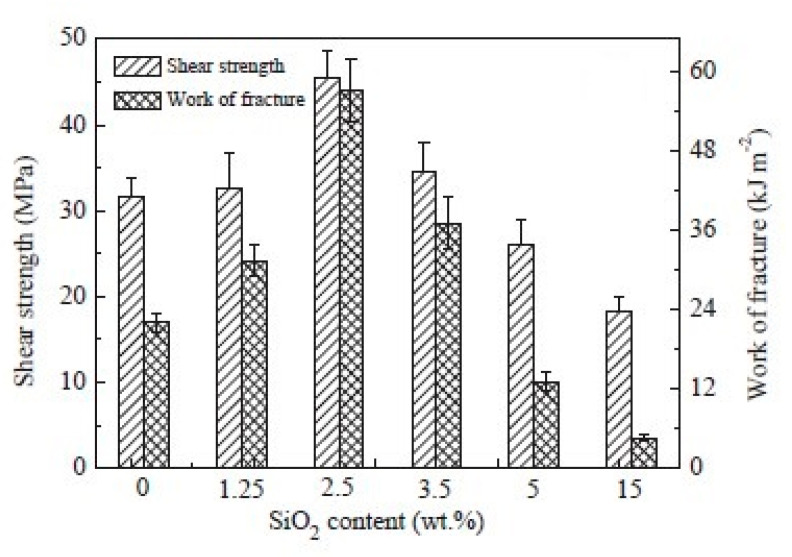
Adhesive strength and work of fracture as a function of nanosilica addition level [44].

The epoxy bonding to titanium alloys, typically for liners in composites pipes for the oil and gas industry, was the subject of the research of Yang et al. [44]. Diglycidyl ether of bisphenol F epoxy resin (DGEBF) blended with an amine hardener was applied in a layer of approx. 0.4 mm to prepare the lap shear test specimen. A silica nanoparticle concentrate in DGEBF was used for the modifications. Bulk strength and toughness of the adhesive were improved with increasing addition levels of nanosilica. Bulk toughness reached a maximum at an addition level of 15 wt %. The lap shear strength of the adhesive joint, however, showed the best performance at 2.5 wt % addition level and was improved by 43%, as can be seen in Figure 12.

In a recent review, several different nanomodifications like nanosilica, nanoclay, nanoalumina, graphene nanoplatelets and carbon nanotubes of epoxy adhesives were compared [45]. Of course, the availability in industrial quantities and the cost associated with the adhesive modification need to be taken into account as well.

Today silica nanoparticles are a well-established raw material for high-performance epoxy adhesives, i.e., for aerospace applications or very demanding automotive bonding applications.

### 3.2. Stereolithography (SLA)

Surface-modified silica nanoparticles are available as concentrates in cycloaliphatic epoxy resins as well and, due to the particle size of 20 nm, appear transparent. Hence, they are very suitable to improve various properties of UV-curable epoxy resin systems. The very low viscosity of such concentrates makes them suitable components in 3D printing formulations.

Of course, most currently used SLA formulations are based on acrylic chemistry. These silica nanoparticles are available in acrylic monomers and oligomers as well, of course with a different surface modification providing acrylic groups on the particle’s surface. To go into details of nanosilica modification of acrylic formulations and the improvements achievable would go beyond the scope of this work. However, some formulations used in SLA applications are combinations of UV-curable epoxy and acrylic resins, and it is intended to provide at least an idea of the possibilities using nanosilica in such applications.

Currently, SLA and other additive manufacturing technologies are maturing from rapid prototyping into rapid manufacturing. This rapidly growing market is highly competitive. As a consequence, not much scientific work regarding the use of silica nanoparticles in such formulations was published, but an abundant number of patents have been filed.

Suriano et al. investigated a modern SLA resin system, based on the combination of cycloaliphatic epoxy resins and acrylic monomers [46]. They reported a reinforcing and toughening effect from the silica nanoparticles. At an additional level of 25.5 wt % nanosilica, the modulus was increased from 2.88 GPa to 3.76 GPa, tensile strength improved from 29.5 MPa to 43.8 MPa (to be compared with a commercial resin having 2.24 GPa of modulus and 29.6 MPa of strength). Shrinkage after one week was extremely low (0.7% compared to 8.5% of the commercial resin) and the surface quality of the printed objects very good. The elongation at break was increased as well by the nanosilica addition, which was considered as increased toughness.

### 3.3. Coatings

As already mentioned in Section 3.2, surface-modified silica nanoparticles are available as concentrates in a large variety of different acrylic monomers and oligomers. These concentrates are used in large volumes in many industrial coating applications, mainly UV-cured acrylic coatings. Examples are parquet coatings or screen coatings. In these applications, the silica nanoparticles provide scratch and abrasion resistance, improve mechanical properties but do not interfere with the color of the coating. Especially suitable they are for transparent coatings, where larger particles, interfering with the visible light, cannot be used. However, epoxy resins are used either in powder coatings or in heavy-duty protective coatings, i.e., for ballast tanks of ships or oil platforms. In such coatings conventional fillers (which are not transparent) are used; often at very high addition levels. Silica nanoparticles do not provide any advantages to these applications, which could not be achieved by using cheaper alternatives.

Subasri et al. investigated using surface-modified silica nanoparticles in combination with gels prepared from silanes and silazanes to coat soda lime glass [47]. They reported good hardness in combination with excellent water repellency and good transparency of the coatings.

A very interesting application was published by White et al. [48]. They used nanosilica in a UV-curable protective epoxy coating formulation, which was released by mechanical damage of the original heavy-duty epoxy coating, thus obtaining a self-healing or regenerative coating system with good mechanical performance.

Zhang et al. recently published a short review regarding nanocoatings and the use of various materials like ZnO, SiO_2_, TiO_2_, CNTs and graphene [49].

Although many patents have been published, unlike acrylic coatings, except for a few niche applications including electronics (which will be covered in Section 3.4), currently silica nanoparticles are not used in epoxy coatings applications.

### 3.4. Electronic Applications

Due to the continuous micronization of electronic components and electronic devices (mobile phones, computers, automotive sensors, etc.) new application areas for silica nanoparticles opened up. With processors becoming smaller but more powerful, more heat is generated which needs to be dissipated. Sockets and connectors become smaller and smaller, but still need to be embedded, which requires epoxy encapsulants with very low viscosities. Nanosilica does not increase the resin viscosity, reduces shrinkage upon cure, stabilizes larger filler particles (often used for heat transfer) and prevents them from sedimentation. Being transparent, they are suitable for fast-processing UV-curable resin formulations. Consequently, soon after the introduction of industrial surface-modified silica nanoparticles into the epoxy resin market, a large number of patents have been filed and are still growing. Contrastingly to stereolithography, over time, more and more research results were published, nevertheless many results of scientific research were never published but directly translated into industrial applications and sometimes protected by patents.

Tanaka et al. investigated the voltage endurance properties of a DGEBA epoxy resin modified with silica nanoparticles and larger, micron-sized fumed silica particles [50]. They measured treeing breakdown time and partial discharge resistance characteristics and found, at identical addition levels, fumed silica to perform better than silica nanoparticles. Both systems were improved in performance by the addition of an epoxy-functional silane.

Bae et al. combined spherical silica nanoparticles and an oligosiloxane resin in a UV-curable cycloaliphatic epoxy resin matrix [51]. They tested this system as a moisture barrier coating for electronic components and found significant improvements for high addition levels (obviously the diffusion path becomes longer) with the transparency of the coating still being very good. In a second step, they tested the best performing formulation as an encapsulant for organic light-emitting diodes (OLEDs) and found the lifetime to be increased by approximately eight times.

A high-performance electronic packaging material, both thermally conductive and electrically insulating was developed by Xue and Xie et al. [52]. They combined silver nanowires and silica nanoparticles in an imidazole-cured DGEBF epoxy resin. Modulus was increased with the nanosilica addition; however, they used a concentrate based on DGEBA, hence modulus is increased further compared to a pure DGEBF system. Thermal conductivity was found to increase by 200% at an addition level of 4 vol % silver nanowires. Adding nanosilica enhanced the thermal conductivity further, up to an increase of 325% at 15 wt % SiO_2_. The electrical resistivity was decreased by the 4 vol % silver nanowires from 1.15 × 10^12^ Ωcm to 6.58 × 10^3^ Ωcm. The addition of silica nanoparticles increased the resistivity, especially for higher loading levels, i.e., 2.62 × 10^12^ Ωcm at 15 wt %.

**Figure 13:**
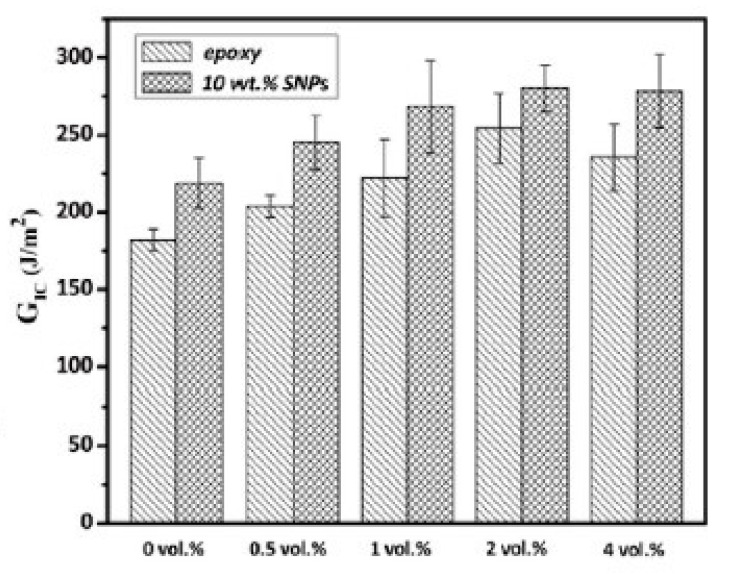
Fracture energy of silver nanowire-modified epoxy resin without and with 10 wt % nanosilica as a function of silver nanowire content [52].

Figure 13 shows the increase in toughness by the addition of 10 wt % silica nanoparticles for different loading levels of silver nanowires. The combination of silver nanowires and silica nanoparticles provided a formulation with excellent mechanical, thermal and electrical properties.

In continuation of their earlier work, Choi et al. conducted an extensive study on a thin-film encapsulant for flexible OLEDs [53]. They optimized the moisture barrier by alternating layers of spin-coated material, which was based on nanosilica containing UV-curable cycloaliphatic epoxy resin. Further details were given in another paper [54]. The same base polymer system was used to design the next generation flexible OLEDs, which exhibited good mechanical reliability and were capable to withstand high temperature/high humidity environments [55].

Renukappa and his team investigated the dielectric properties of a CTBN-toughened DGEBA epoxy resin with various addition levels of nanosilica [56]. The dielectric constant increased with increasing SiO_2_ content with an optimum at 10 wt %. Diffusion of water into the epoxy during seawater aging was delayed (reduced weight gain), but similar to unmodified epoxy after 96 h. The highest dielectric constant was achieved under seawater aging conditions again with 10 wt % nanosilica.

An extensive review of the use of various nanoscaled fillers including nanosilica to optimize epoxy resins for high voltage insulation was published by Einarsrud et al. [57] which can help to dive deeper into the subject.

An anhydride-cured epoxy resin was studied by Bajpai et al. [58,59]. This resin system was modified with a core shell rubber Type I and silica nanoparticles using a concentrate in DGEBF. The fracture toughness of the neat epoxy resin was increased from 0.10 kJ/m^2^ to 0.48 kJ/m^2^ adding 10 wt % nanosilica, respectively, to 0.75 kJ/m^2^ by adding 5 wt % core shell rubber. The hybrid system with both 5 wt % core shell rubber and 10 wt % nanosilica performed best with a G_Ic_ of 1.01 kJ/m^2^ (compare results from [24]). A modification of the neat resin with 0.075 wt % carbon nanotubes increased the conductivity from approx. 10^−14^ to 10^−17^ S/cm to 2.6 × 10^−4^ S/cm. However, strength was lowered from 86 MPa to 72 MPa; toughness was minimally increased to 0.18 kJ/m^2^. The combination of 0.075 wt % CNT and 10 wt % SiO_2_ provided a similar conductivity 2.2 × 10^−4^ S/m), but a strength of 84 MPa and a toughness of 0.72 kJ/m^2^.

### 3.5. Fiber-Reinforced Composites

Fiber-reinforced composites are currently the application consuming by far the largest volumes of silica nanoparticles in epoxy resins. Consequently, quite a few researchers are working in this field, as well as many patents have been filed. The extremely small size of silica nanoparticles compared to fiber diameters enables them to penetrate even close-meshed fabrics when being infused or injected. Even over long distances no gradients in nanosilica concentrations were found (compare [60]). Louis et al. thoroughly investigated an amine-cured DGEBA containing silica nanoparticles in an RTM process using a twill weave aramid textile and found no filtration to occur during the infusion process [61].

**Figure 14:**
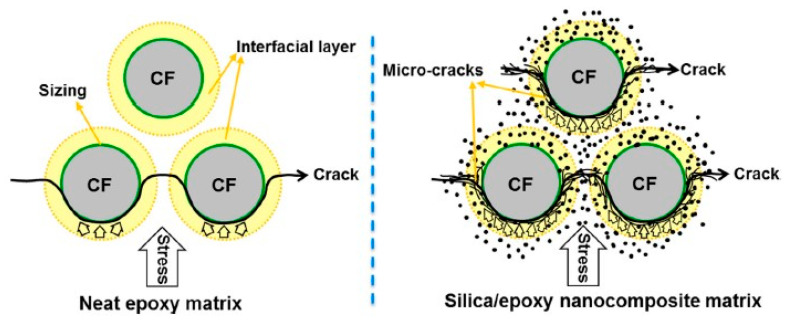
Schematic illustration of energy dissipation and stress transfer in the interphase layer of FRCs for an unmodified epoxy and a nano-modified epoxy matrix [62].

Zhang et al. investigated the influence of silica nanoparticles on the interfacial properties of an anhydride-cured DGEBA epoxy resin reinforced with carbon fiber bundles to simulate a unidirectional (UD) laminate [62]. They reported an increase in interlaminar shear strength (ILSS) by 13% at a 10 wt % addition level of nanoparticles. They concluded that the matrix was toughened by the nanoparticles by reducing the stress concentration at the fibers and dissipating deformation energy, thus slowing down the crack formation between carbon fiber and epoxy resin matrix, as can be seen in Figure 14.

**Figure 15:**
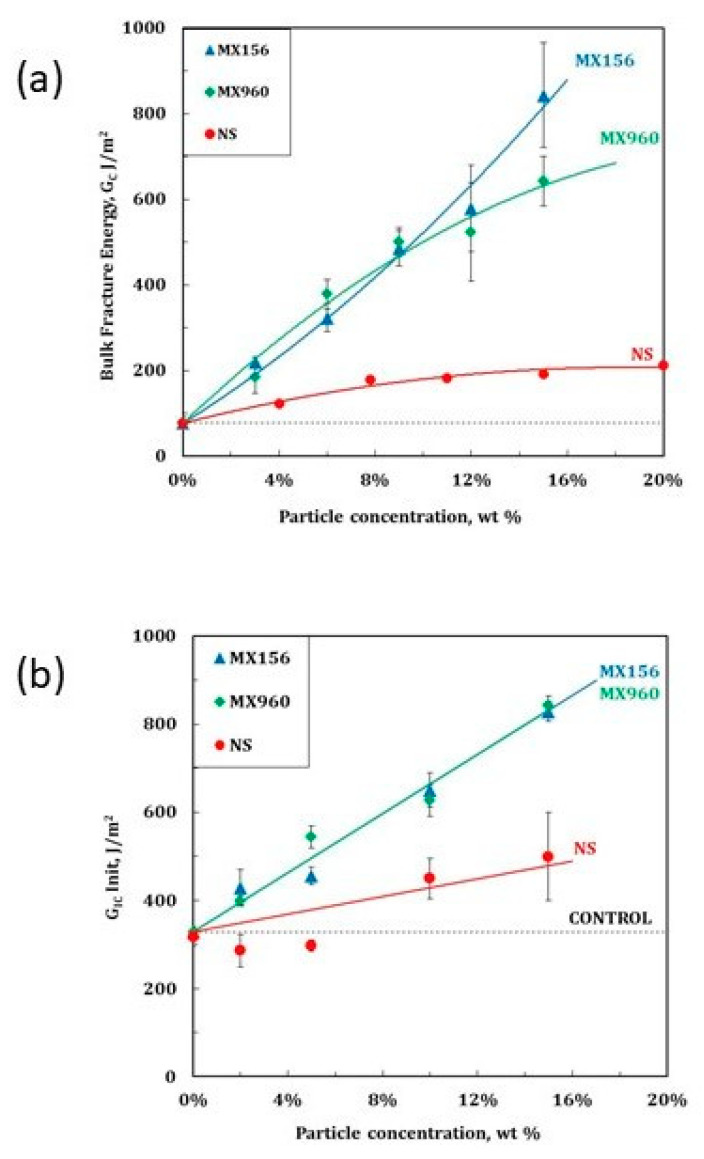
Fracture energy versus particle addition level for bulk (**a**) and glass fiber-reinforced composite (**b**) [63].

Taylor et al. manufactured various laminates using a multiaxial ±45 ° glass fiber fabric and an anhydride-cured DGEBA in a vacuum-assisted resin infusion (VARI) process [63]. They used silica nanoparticles and two different core shell rubbers Type I (polybutadiene core and polysiloxane core) to modify the epoxy resin. The fracture toughness increased with increasing nanosilica addition levels. Compared to the classic micron-sized elastomeric core shell tougheners the improvements are minor, however. Similar findings were reported in earlier work [60]. The modification with nanosilica does not provide significant improvements in laminate toughness; although other properties like fatigue performance upon cyclic loading are increased significantly. In Figure 15, the toughness improvements achieved by the different modifications are shown for the bulk epoxy resin (Figure 15a) and the glass fiber-reinforced composite (GFRC) (Figure 15b).

In continuation of this work, Taylor et al. had a close look at a hybrid resin system, an anhydride-cured DGEBF, which was modified with 9 wt % rubber toughener (CTBN) and 10 wt % nanosilica [64]. Glass fiber-reinforced laminates were made using ±45° biaxial textile and a VARI process. The G_Ic_ of the unmodified control was determined to be 1.09 kJ/m^2^, which was increased by a nanosilica-only modification to 1.31 kJ/m^2^ and by a CTBN-only modification to 1.83 kJ/m^2^. The hybrid resin system with both modifications had a G_Ic_ of 1.65 kJ/m^2^. A synergy was found between the two modifications regarding threshold fracture energy, which was greatly improved.

Tate et al. worked on a low-velocity impact study testing laminates made by using a ±45° biaxial glass fiber fabric in a VARTM process [65]. The amine-cured DGEBA was modified with 6.5 wt % CTBN and 8.1 wt % silica nanoparticles.

The compressive strength after impact was found to be reduced for the hybrid, this is probably due to the superior impact energy dissipation of the modified system. The backside delamination is decreased, a very important property in ballistic applications where resistance to penetration is more important than the minimization of the damaged area. Similar behavior was found for carbon fiber-reinforced laminates [66].

**Figure 16:**
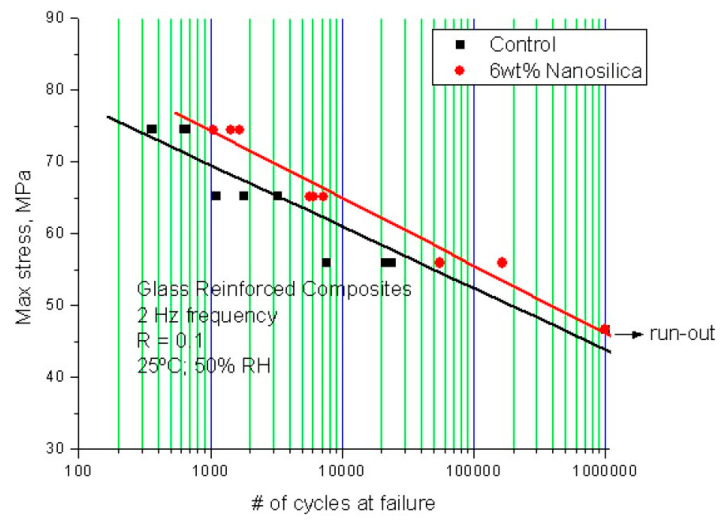
Stress versus number of cycles until failure (S–N) curves for unmodified and 6 wt % SiO_2_-containing epoxy GFRC [67].

In another study Tate et al. investigated a typical system for rotor blades for wind energy generators [67]: a ±45° multiaxial glass fiber fabric was impregnated by an amine-cured DGEBA using a VARTM process. A modification with 6 wt % silica nanoparticles increased both tensile and flexural modulus, tensile and flexural strength as well as ILSS. Most importantly, however, was the improved fatigue performance of the laminates, as can be seen in Figure 16.

Landowski and Imielinska researched the influence of water exposure on glass fiber-reinforced laminates made by VARTM using an amine-cured DGEBA [68]. The impact damaged area of the laminates modified with 3 wt % nanosilica was larger than for the unmodified control. After water absorption >1% the damaged area of the SiO_2_-modified became smaller compared to the control, they assumed due to an increased nanoparticle debonding caused by the water uptake.

Two resin systems, anhydride-cured DGEBA and DGEBF were modified with silica nanoparticles (concentrate in DGEBF) and carbon fiber bundles (12 K) were used to manufacture the test specimen to model UD laminates by Jiang and Huang et al. [69]. They reported improved strength and modulus of the bulk resins. The transverse tensile strength of the laminates was improved by the nanosilica addition as well.

Tang et al. used a VARTM process to make carbon-fiber-reinforced UD laminates based on amine-cured DGEBA [70]. A modification with 2 wt % nanosilica increased the laminate G_Ic_ by 17% and the ILSS by 8%. The addition of 3 wt % polydopamine increased G_Ic_ and ILSS further. The test specimen was subjected to salt spray exposure for one week, respectively, for three weeks. This reduced both G_Ic_ and ILSS, but as the modified laminates started at higher values, they still performed significantly better after the salt spray exposure than the control.

Mahrholz et al. researched the influence of process parameters on the surface quality of CFRCs [71]. They manufactured laminates using both a satin weave and a UD carbon fiber textile in an RTM process. The amine-cured DGEBA was modified with silica nanoparticles. They found a reduced volume shrinkage (equals an improved laminate surface) for a precure at lower temperatures. Modification with nanosilica improved bulk resin strength and modulus nearly linear. The coefficient of thermal expansion (CTE) was reduced with increasing SiO_2_ addition below and above the glass transition temperature (T_g_). Bulk volume shrinkage was reduced by 75%. Combining the nanoparticle addition with the optimized cure cycle provided laminates with Class A surfaces.

Sprenger et al. investigated the properties of a CFRC made by VARTM using a ±45° biaxial textile and an amine-cured DGEBA [66]. The system modified with 10 wt % CTBN and 10 wt % nanosilica showed the same modulus as the unmodified control, but G_Ic_ was increased by 250%. Compressive strength after impact was lower than for the control; the delaminated area was larger. This indicates the excellent impact energy dissipation of the laminate with the hybrid epoxy resin.

Abliz et al. used a quasi-unidirectional carbon fiber fabric and an anhydride-cured DGEBA for their studies [72]. Flow behavior of epoxy resins modified with silica nanoparticles and boehmite nanoparticles was studied. They could show that with an increasing fiber volume fraction the particle size distribution and the dispersion of the nanoparticles become increasingly important. Further details can be found in the thesis the paper was based on [73].

A plain weave carbon fiber fabric was infused by VARI with an amine-cured DGEBA from Landowski and his team [74]. They tested various properties and interestingly reported a decrease in damage area by 27% for an epoxy resin modified with 8 wt % silica nanoparticles. This seems to be in disagreement with the results of others, as previously reported. However, it simply shows the extreme importance of the textile used as reinforcement when it comes to impact testing. Depending on the fiber orientation towards the direction of the impact the engineer can achieve quite different mechanical behavior by changing the design.

Kinloch et al. studied the mechanical and fracture performance of an anhydride-cured DGEBA which contained an aliphatic epoxy (HDDGE) as well [75]. A biaxial ±45° carbon fiber fabric was used in a VARI process to manufacture the laminates to be tested. The epoxy resin was modified with silica nanoparticles and core shell particles Type III. The fracture toughness was determined at 20 °C and at −80 °C. The unmodified control showed a G_Ic_ of 0.17 kJ/m^2^ at RT and 0.15 kJ/m^2^ at −80 °C for bulk and a G_Ic_ of 1.25 kJ/m^2^ at RT and 0.87 kJ/m^2^ at −80 °C for the fiber-reinforced composite. The modification with 8 wt % nanosilica increased the fracture toughness by 15% in bulk and by 5% in the composite, whereas a modification with 8 wt % core shell elastomer showed improvements of 440%, respectively, 35%. The best performance was again found for the hybrid resin system with 8 wt % SiO_2_ and 8 wt % core shell having a G_Ic_ of 1.22 kJ/m^2^ in bulk and 1.76 kJ/m^2^ for the composite, which equals improvements of 600% and 40%. At −80 °C the G_Ic_ of the composite control was lowered by 30%, the laminate made with the hybrid resin system lost only 23%. The main toughening mechanisms were localized plastic shear-band yielding initiated by the nanoparticles and plastic void growth around cavitated core shell elastomer particles.

In continuation of this work, Carolan et al. predicted fracture energies by modeling the fracture mechanisms and found good agreement with the experimental data [76] for both test temperatures.

In a very different approach, Tsai et al. investigated the toughness properties of carbon fiber-reinforced composites made by using carbon fibers which had a sizing containing silica nanoparticles [77]. The sizing, a thin coating usually applied during fiber manufacturing protects the fibers during processing and weaving and improves adhesion between fiber and matrix upon curing of the composite. As a matrix, they used an anhydride-cured DGEBA. Fracture toughness tests showed an increase in G_Ic_ of 33% of the laminates, although the amount of nanosilica (introduced by the sizing and localized on the fiber) was less than 1 wt %. This indicates the importance of the fiber–matrix interphase (compare the suggested stress distribution mechanisms [62]), providing and elegant and low-cost method to improve laminate toughness. However, no significant improvements in fatigue performance were found.

Recently, Zhang et al. published a review comparing the improvements of both glass and carbon fiber-reinforced composites by the use of various silica nanoparticles [78]. They concluded that the nanosilica can greatly improve the mechanical properties as well as other important properties of laminates and confirmed the suitability of nanosilica for all current composites manufacturing methods. Furthermore, they pointed out the importance to achieve a very good dispersion of the nanosilica in the epoxy resin in order to obtain the desired improvements.

**Figure 17:**
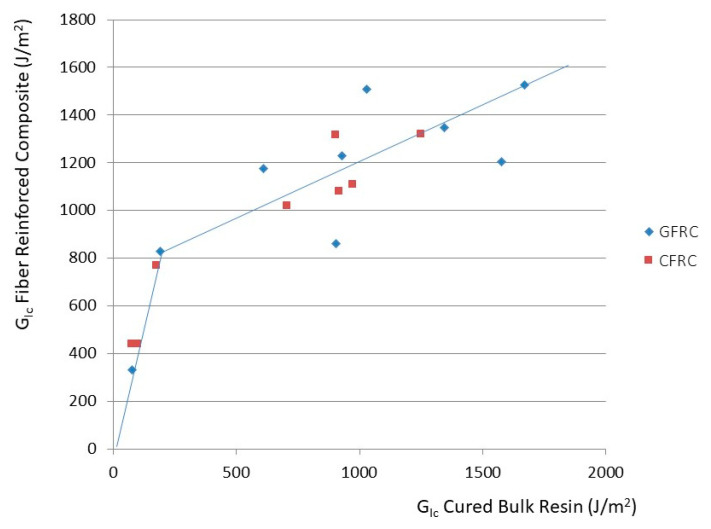
Bulk G_Ic_ versus laminate G_Ic_ of modified epoxy resins [79].

In another review, it was found that regardless of the reinforcing fibers and their weave a relationship between the fracture energy of the bulk epoxy resin matrix and the laminate exists [79]. The percentage increase in G_Ic_ achieved for an epoxy resin by the modification with silica nanoparticles and an elastomeric toughener in the bulk resin improves the G_Ic_ of the corresponding laminate by the transfer factor 0.18. This helps the engineer to design the matrix resin: if an improvement of 30% in laminate fracture toughness is necessary, the epoxy resin used needs to be replaced by a nano-modified resin having a G_Ic_ 170% higher than the previous resin. Figure 17 shows the G_Ic_ of bulk resin systems modified with silica nanoparticles and an elastomeric toughener versus the G_Ic_ of laminates made thereof (both glass and carbon fiber-reinforced). The data makes evident why such resin systems are currently used in large volumes in the composites industry.

**Figure 18:**
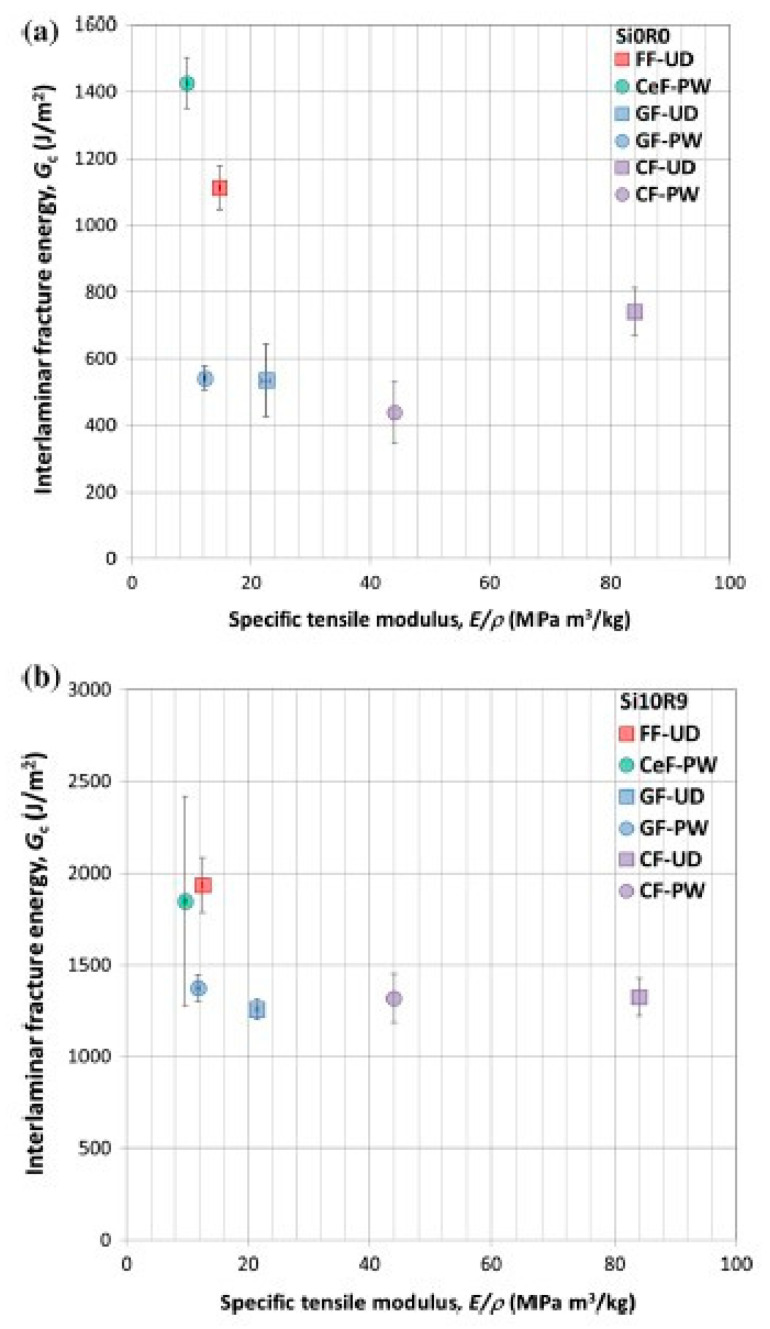
Fracture energy versus modulus for various laminates with unmodified epoxy resin and epoxy resin modified with 10 wt % nanosilica (**a**) and 9 wt % CTBN (**b**) [80].

Natural fibers as reinforcement of composites are becoming more and more of interest, given the need to switch to renewable raw material sources and towards more sustainable products. Kinloch et al. investigated an anhydride-cured DGEBA matrix system, modified with silica nanoparticles and a reactive liquid rubber (CTBN) [80,81]. A VARI process was used to make the laminates based on two natural fiber systems: a UD flax fiber fabric and a plain-woven regenerated cellulose fiber fabric. These laminates were compared to laminates reinforced with traditional glass fiber fabrics. Of course, it is extremely important to predry the natural fibers directly before the infusion process as they tend to pick up moisture upon storage, which leads to laminates with inferior properties. It was found that both CTBN and nanosilica did increase the laminate toughness, but that the hybrid epoxy resin containing both modifications performed best. In Figure 18, the G_Ic_ values versus the modulus for composites made with the unmodified epoxy resin (Si0R0) and the hybrid epoxy resin with 10 wt % nanosilica and 9 wt % CTBN (Si10R9) are shown. It is important to note the different scales of the G_Ic_ on the *y*-axis.

The abbreviation FF stands for flax fiber, CeF for cellulose fiber, GF for glass fiber and CF for carbon fiber. UD for unidirectional and PW for plain weave. It is evident that the laminates based on natural fibers provide by far the toughest laminates, whereas their modulus is similar to a plain weave glass fiber fabric. It is obvious that natural fibers cannot compete in modulus with carbon fibers. However, the combination of very high fracture toughness and good mechanical properties makes them very attractive for industrial applications, i.e., automotive parts. Again, the modification of the epoxy resin matrix with an elastomeric toughener and silica nanoparticles improves laminate performance significantly (compare different *y*-axis in Figure 18).

## 4. Conclusions and Outlook

The rheological properties of uncured epoxy resin systems are not or nearly not affected by the modification with silica nanoparticles. This is very important for all manufacturing processes where viscosity is critical. The nanoparticles do reduce the reaction heat, hence enabling the use of very fast cure cycles which equal high productivity.

Using commercially available nanosilica concentrates in epoxy resins and respecting the safety precautions required by the handling of epoxy resins does not create health risks in production nor in the use and recycling of the epoxy resin.

The modification of epoxy resins with silica nanoparticles does improve various properties of cured bulk resins, like modulus, toughness, compressive properties, reduced CTE, increased fatigue resistance. The T_g_ remains largely unaffected.

The toughening mechanisms are partial debonding of silica nanoparticles followed by plastic void growth and localized shear banding.

The combination of conventional, micron-sized elastomeric tougheners and silica nanoparticles provides epoxy resin formulations with outstanding performance: very tough, high modulus, outstanding fatigue performance, etc.

Depending on the application of the modified epoxy resins, besides these general property improvements specific application-related properties can be improved by the addition of silica nanoparticles; i.e., the scratch resistance of coatings, reduced electrical corrosion of electronic encapsulants, Class A surface of fiber-reinforced composites, outstanding fatigue performance of adhesives.

Taking into account the nanosilica particle development in the last 20 years, it is very unlikely that a new type of nanosilica will be commercialized in the coming years. Various manufacturers disappeared and spherical particles with various sizes have been taken off the market. Eventually, different surface modifications optimized for special applications might arise.

The great potential for further significant improvements of epoxy resin properties and the respective industrial applications lies in the combination of different modifications. As could be seen with the combination of µm-sized elastomeric tougheners and nanosilica (compare Section 2.4), the mechanisms of toughening can be synergistic. This could be the case for other property improvements as well. An example of such future combinations could be an epoxy resin modified with nanosilica and graphene, spherical particles and platelets as combination. Another area currently of high interest are flame-retarded epoxy resin systems, where nanosilica could push the performance of other resin modifications.
